# Assessment of Human Health Vulnerability to Climate Variability and Change in Cuba

**DOI:** 10.1289/ehp.8434

**Published:** 2006-07-11

**Authors:** Paulo Lázaro Ortíz Bultó, Antonio Pérez Rodríguez, Alina Rivero Valencia, Nicolás León Vega, Manuel Díaz Gonzalez, Alina Pérez Carrera

**Affiliations:** 1 Climate Center, Institute of Meteorology, Havana, Cuba; 2 Tropical Medicine Institute “Pedro Kourí,” Havana, Cuba; 3 Vector Control Department, Ministry of Public Health for Cuba, Havana, Cuba

**Keywords:** climate change, climate indices, climate variability, human health, impacts

## Abstract

In this study we assessed the potential effects of climate variability and change on population health in Cuba. We describe the climate of Cuba as well as the patterns of climate-sensitive diseases of primary concern, particularly dengue fever. Analyses of the associations between climatic anomalies and disease patterns highlight current vulnerability to climate variability. We describe current adaptations, including the application of climate predictions to prevent disease outbreaks. Finally, we present the potential economic costs associated with future impacts due to climate change. The tools used in this study can be useful in the development of appropriate and effective adaptation options to address the increased climate variability associated with climate change.

Concern about the potential health efffects of climate change began in the mid-1980s, with indications that emission of greenhouse gases from human activities could influence the climate system and result in intensification of the greenhouse effect. Given the clear evidence that many health outcomes are highly sensitive to climate variations, it is inevitable that long-term climate change will have some effect on global population health. Climate variability (CV) and change will influence all natural, human, and socioeconomic systems, thus affecting not only health but also many aspects of ecologic and social systems. Climate is one factor that may create conditions that facilitate the development of some disease-causing microorganisms (McMichael and Kovats 1999)

It is important for the health sector to understand current vulnerability to CV because this increasing variability may have a greater impact on health than gradual changes in mean temperature, precipitation, and other climatic variables. Assessing current vulnerability includes understanding both disease exposure–response relationships and current interventions implemented to reduce the burden of climate-sensitive diseases. Additional interventions that can be implemented within the time frame of decision makers (5–10 years) need to be identified to reduce the health effects projected to occur with climate change.

Climate-sensitive diseases have been identified that have important health burdens, particularly vectorborne diseases. Virus and bacteria quickly mutate, thus allowing for environmental adaptation (McMichael and Kovats 1999). CV and climate change may be additional stresses that increase mutation rates of different microorganisms, thus increasing emerging and reemerging diseases. Climate is not the only factor that affects the incidence and range of vectorborne diseases; recent increases are due at least in part to the collapse of vector-control programs (Burton and van Aalst 1999; Michael and Trtanj 1999).

We included acute respiratory infections (ARIs), acute diarrheal diseases (ADDs), bacterial meningitis, viral meningitis, dengue fever, and bronchial asthma (BA) in the vulnerability assessment because these diseases are known to be climate sensitive and because they have relatively high burdens of disease in Cuba.

We analyzed interactions between CV and disease burdens, taking into account that epidemic processes are multicausal (Chan et al. 1999; Kovats et al. 2003). We also explored the uses of a climate index in the prediction of disease outbreaks (Michael and Trtanj 1999; World Meteorological Organization 2001). We then synthesized this information to describe current vulnerabilities to CV, including descriptions of the adaptation baseline. Finally, we estimated the potential economic costs of the projected health impacts of climate change.

## Materials and Methods

### Data sources

#### Weather and climate data

We obtained meteorologic data from 1961 to 2003 from the Climate Center at the Institute of Meteorology; data are available from 52 stations across the country. Data included monthly series of maximum and minimum mean temperatures (°C), precipitation (millimeter), atmospheric pressure (hectopascal, i.e., 10^−2^ pascal), vapor pressure (millimeter of mercury), percent relative humidity (percent), thermal oscillation, days with precipitation, solar radiation in (mega-joules per square meter), and isolation (hours of light). The period 1961–1990 constituted the climate baseline.

Three monthly variables were included to account for interannual and decadal variability: multivariate ENSO (El Niño Southern Oscillation) index (MEI), quasi-biennial oscillation (QBO), and North Atlantic Oscillation (NAO). Data were available since 1950 from the Climate Diagnostic Center.

#### Epidemiologic data

The Ministry of Public Health provided epidemiologic data that were obtained from the National Statistical Branch for 1961–2003, including the number of cases and rate of primary health care visits for ARIs, ADDs, viral hepatitis (VH), varicella (chicken pox), meningococcal disease, meningitis caused by *Streptococcus pneumoniae*, and *Plasmodium falciparum* and *Plasmodium vivax* malaria.

#### Ecologic data

We obtained an ecologic database from the Unit of Fight and Vector Control in the Ministry of Public Health. This database contained information from 1981 to 2004 on its *Aedes aegypti* [vector for yellow fever, dengue fever, and dengue hemorrhagic fever (DHF)] monitoring and surveillance system. Data included the larval density, biting density per hour of the vector, and the positive house index, which is the ratio of the number of houses positive for larvae to the number of houses inspected.

#### Socioeconomic data

We obtained data from the National Planification Center from 1981 to 2003, including the percentage of houses without potable water, percentage of houses with dirt floors, the adult (≥ 16 years of age) illiteracy rate, monthly bird rates, and an index of monthly infestations of *A. aegypti* based on the number of houses where a foci of *A. aegypti* mosquitoes was observed.

### Statistical methods

The empiric orthogonal function (EOF) analysis method has been used extensively in meteorologic and climatologic studies [for more information on EOF, see Hair et al. (1999)]. The EOF is designed to derive the dominant variability patterns from sets of fields of any type, synthetic indicators or indexes, and summarize the variability observed in a group of variables. This method facilitates the description of associations between the weather or climate, CV, and health outcomes (Basilevsky 1994; Dillon and Goldestein 1984; Lorenz 1956). Uncertainties with this method include the use of the scale additive and the factor score. The approach used will depend on the objectives, for example, if one wants to achieve a measure that maintains the condition orthogonally (factor score) and that is applicable to other studies (scale additive). The scale additive creates a compound or synthetic measure using substitute variables, with those variables that contribute more weight for each selected factor acting as representatives of related factors. The weight values for each component were used to obtain the indices that represent the main source of variation in climate information according to the following equation:


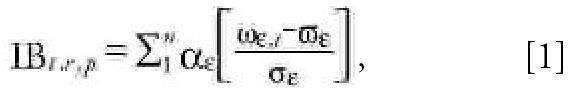


where IB*_t_*_,_*_r_*_,_*_p_* is the Bultó index, which expresses the variability at time *t*, in region *r*, in the country *p*; ɛ describes the CV that characterizes the study region; α_ɛ_ is the weight for each variable; ω_ɛ,_*_t_* represents the weather and CV series at time *t*; ωɛ is the mean value of the weather and CV; and σɛ is the standard deviation of the variable. According to Equation 1, the anomalies in the different scales of the variability are expressed by linear combinations of variables that contributed the most to each component (Ortíz Bultó and Rivero 2004; Ortíz Bultó et al. 1998, 2000).

Four indices were constructed to describe climate trends and variations that influence ecosystem dynamics, leading to changes in the incidence of climate-sensitive diseases. These indices were used to describe the relationships between climate and health in Cuba (Ortíz Bultó et al. 2000):

- IB*_t_*_,1,_*_c_* describes intermonthly and interseasonal variation; it includes maximum and minimum mean temperature, precipitation, atmospheric pressure, vapor pressure, and relative humidity.
- IB*_t_*_,2,_*_c_* describes seasonal and interannual variation; it includes solar radiation and sunshine duration as factors that affect temperature and humidity. Positive values are associated with a high solar energy level.
- IB*_t_*_,3,_*_c_* describes interannual and decadal scale variation; it includes the same climate variables as IB*_t_*_,1,_*_c_*.
- IB*_t_*_,4,_*_c_* describes the relationships among socioeconomic variables and can be interpreted as life quality or the degree of poverty as they influence disease risk.

Other factors that influence disease transmission include the abundance and geographic distribution of the vector, as well as the socioeconomic conditions and trends. In the case of socioeconomic data, similar procedures were used to construct an indicator.

### Cuban climate

The Cuban archipelago comprises the island of Cuba, the Juventud Island, and 1,600 small islands and keys. The Cuban climate results from its location in the northern portion of the tropics, near the Tropic of Cancer. The climate changes little over the year. Cuba’s climate is tropical and seasonally wet, with marine influence and semicontinental features. The months from May to October are generally hot and rainy, and those from November to April (winter, the dry season) are characterized by lower ambient temperatures and precipitation. The rainfall depends on the intensification or weakening of the North Atlantic subtropical anticyclone. The most important changes are linked with the presence of disturbances in the tropical circulation (tropical waves and hurricanes). Tropical cyclones also contribute to total rainfall.

In winter, drought conditions can be severe in almost all parts of Cuba, particularly in the eastern region. Drought reduces the water available for washing and sanitation and increases the risk of disease.

#### The effect of El Niño in Cuba

CV can be expressed at various temporal scales (by day, season, and year). The ENSO has been a significant element contributing to CV in Cuba. In Cuba, ENSO events cause significant anomalies in atmospheric circulations patterns, resulting in positive rainfall anomalies and increased minimum temperatures during the winter months (dry season) and an increased frequency of severe weather events (Cárdenas 1998).

Many regions can be affected when rainfall increases by an increase in vector density and transmission potential (McMichael et al. 2003). Ecosystem effects are significant, resulting in high levels of *A. aegypti*. Temperature also affects the behavior of the vector and humans, increasing the probability of transmission (e.g., increases in temperature decrease the incubation period of the mosquitoes). The numbers of cases of diarrhea also increase considerably because of poor sanitary conditions.

#### Climatic trends in Cuba

Since about the 1950s, the mean ambient temperature in Cuba increased between 0.4 and 0.6°C. Minimum temperatures increased approximately 1.5°C, whereas the maximum temperature remained almost constant. These warmer temperatures were associated with an increase in winter precipitation and a decrease in summer precipitation. The increase in winter precipitation can be linked to an increase in the frequency of extreme events, particularly after the 1970s. The main climate trends observed in Cuba during the 1990s include a decrease in the diurnal temperature range by 2°C; an increase in precipitation in the dry season and a decrease in the wet season; a later start of the wet and dry seasons, with a lag in summer precipitation; an increase in extreme weather events, such as droughts, floods, and other dangerous meteorologic events; stronger hurricane seasons; and more frequent extreme events, particularly ENSO (warm events in 1991–1993–1994–1995–1997–1998, and 2002–2003, and cold events in 1994, and cold events in 1996, and cold events in 1998–1999, and 1999–2000).

The frequency of climate anomalies increased in the last decades. The Climate Center at the Institute of Meteorology has a prediction model of the multivariate ENSO index (PMEI) that forecasts the occurrence of El Niño or La Niña events 3 months in advance (Ortíz and Rivero 2003b). Positive values are associated with warmer events and negative values are associated with cold events.

**Figure 1:**
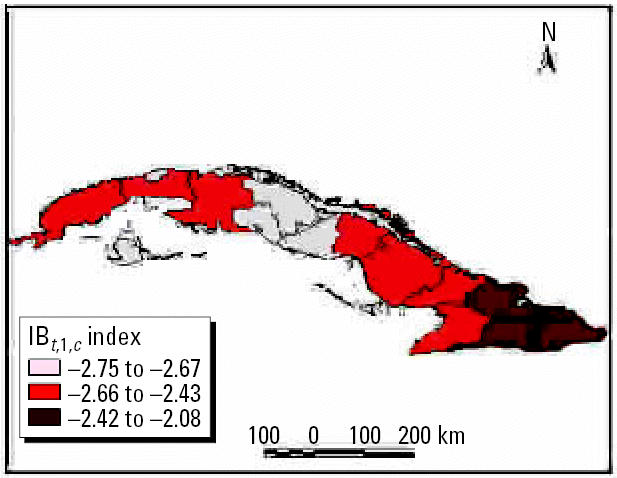
Winter trend anomalies in the 1980s using the IB*_t,_*_1_*_,c_* index.

**Figure 2:**
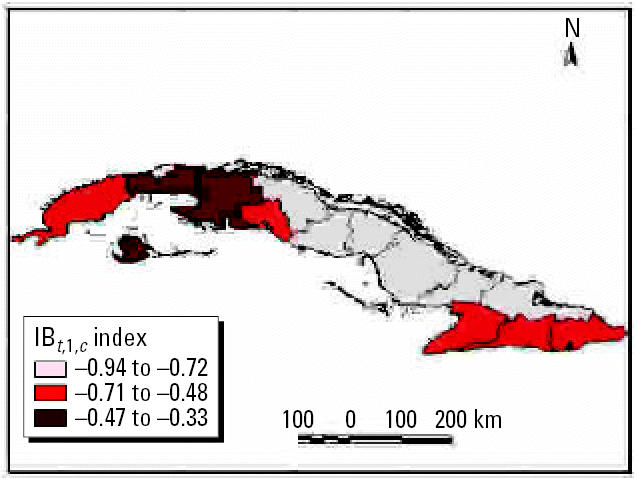
Winter trend anomalies in the 1990s using the IB*_t,_*_1_*_,c_* index.

Winter trend anomalies in the 1980s and 1990s are shown in Figures 1 and 2. These figures show the warmer conditions and increases in drought in the eastern region.

## Results and Discussion

### Priority climate-sensitive diseases in Cuba

Public health is a high priority in Cuba. Reliable disease surveillance began in 1960. In 1997 the most prevalent diseases were ARIs, ADDs, BA, VH, and chicken pox; rates were 43,905, 8,997, 8,200, 239, and 223 per 100,000, respectively. The prevalence of BA was 8.6% in urban areas and 7.5% in the rural zones. Other important diseases include gonorrhea and syphilis, with rates of 304 and 142 per 100,000. There were low rates of meningococcal (3 per 100,000), bacterial (9 per 100,000), and viral meningitis (26 per 100,000). There were no reported cases of poliomyelitis, diphtheria, whooping cough, measles, rubella, mumps, or neonatal tetanus as a result of vaccination programs carried out since the early 1960s. In 1998 the Health National System reported there were 1,783 medical care institutions offering medical assistance to 100% of the population (Gutiérrez 1998).

Dengue fever was first identified in Cuba in 1943, although it may have caused an epidemic in 1902. In 1977, dengue serotype 1 was introduced and quickly spread throughout the country. During the resulting epidemic, which lasted to 1978, 553,132 cases were reported. The first great epidemic of DHF in the Western Hemisphere occurred in Cuba in 1981, with 344,203 cases of dengue fever, 10,312 of DHF, and 158 deaths (Guzmán et al. 1990; Kourí et al. 1989, 1997). Dengue serotype 2 was the causative agent. In response, a vector control program was initiated with support from all levels, including direct actions by the president of the Cuban government. In addition a surveillance program was implemented, including the establishment and improvement of diagnostic laboratories. These programs had good results, with no autochthonous cases reported until 1997; Cuba was declared free of *A. aegypti*, except in the capital (Havana, population of 2.25 million) and the cities of Santiago de Cuba and Guantánamo. Since the initiation of the vector-control programs, *A. aegypti* has been detected (and quickly eliminated), particularly along the highway that unites these cities. The main difficulties in Havana have been the size of the city and the population heterogeneity. In Santiago and Guantánamo the primary problem has been the lack of a constant supply of drinking water, which compels the population to store water in containers that serve as breeding sites for the vector. As a result, these cities have experienced epidemics in recent years. In 1997, Santiago de Cuba was affected by an epidemic in which 17,114 clinical cases were reported, including 205 cases of DHF and 12 deaths (Kourí et al. 1997). The next epidemic was in 2000 in Havana, where there were 138 cases of dengue. Another epidemic occurred in 2001–2002 in Havana, with nearly 12,000 cases.

VH type A is a water- and foodborne disease that is highly resistant to extreme environmental conditions, contributing to viral persistence and the possibility spreading throughout the community (Piatkin and Krivochein 1981). In Cuba VH type A is seasonal, increasing from August to October during the baseline period 1961–1990. However, with recent CV and climate change, winter seasons are becoming warmer and rainier (greater CV), resulting in an advance of the peak months of transmission to March through June of each year. These new seasonal conditions are shown by the range of values of the indices IB*_t_*_,1,_*_c_* and IB*_t_*_,3,_*_c_* ; IB*_t_*_,1,_*_c_* is highly positive and values of IB*_t_*_,3,_*_c_* are moderately positive.

**Figure 3:**
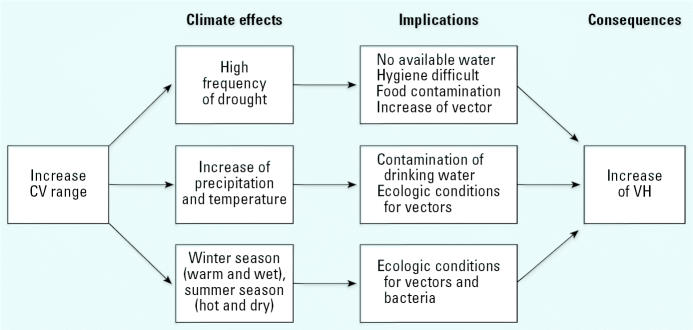
Relationship between CV and VH.

These climatic patterns favor contamination of drinking water due to wastewater or overflow, resulting in a quick increase in vectors such as flies and cockroaches when poor sanitary conditions are combined with warm and humid conditions (Figure 3). Although different specific agents are involved, similar behaviors and mechanisms are observed with diarrheal diseases. Climate anomalies can increase the incidence of waterborne diseases; this is most likely to occur within communities that do to not have adequate drinking water supplies and sanitation systems (McMichael et al. 1996).

There are multiple causative agents of ARIs, with the most frequent being those of viral origin. Drought, cold winds, and abrupt temperature variation during the winter season, combined with an increase in dusty conditions, can insult the mucous membranes of the respiratory passages, which can facilitate contracting an ARI (San Martín 1963). In addition, close personal contact during winter months can contribute to the spread of ARIs.

**Figure 4:**
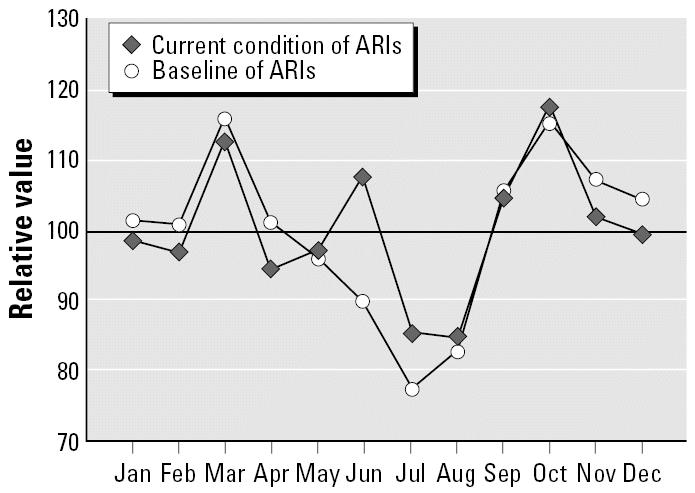
Impact of CV on the seasonal pattern of ARIs.

During the 1961–1990 baseline, peaks in March and October characterized ARIs (Figure 4). Currently, as a consequence of increasing climate anomalies (e.g., drought and warmer winters), a new peak is now observed in June when the rainy season is delayed (Figure 2). Low temperatures during the winter season and close contact among persons also may be possible causes of this increase. These changes are shown in the response of the combination of the climatic indices IB*_t_*_,1,_*_c_* and IB*_t_*_,2,_*_c_*, with a high range of IB*_t_*_,1,_*_c_* and a low range of IB*_t_*_,2,_*_c_* characterizing warmer and drier summer seasons.

Chicken pox is transmitted person to person. During the baseline period the seasonal peak was in March (the end of winter). Currently, the peak is observed in April, a month characterized by high CV. High CV may result in insults to the upper respiratory tract, increasing viral transmission, particularly among infants and children. The climate patterns are characterized by a combination of moderate IB*_t_*_,1,_*_c_* values with high IB*_t_*_,3,_*_c_* values (dry and high contrasting conditions).

The main cause of bacterial meningitis in Cuba since 1999 has been *S. pneumoniae*, a bacterial agent common in the upper respiratory tract. CV apparently contributes to the infection, particularly in children younger than 5 years and the elderly. The disease occurs most often during January to April, but there is a regional difference in the pattern of *S. pneumoniae*. The central region has higher solar radiation and more CV, which is shown by high IB*_t_*_,2,_*_c_* values and low IB*_t_*_,3,_*_c_* values. The combined physical–geographic characteristics and socioeconomic conditions (IB*_t_*_,4,_*_c_*) may explain the high incidence of disease.

Some authors (Pan American Health Organization 1997) suggested that pneumococcal infections might increase in the winter. In Cuba the number of weekly cases increase approximately 4-fold from summer to winter, and a prominent peak in the number of weekly cases occurs during the last week of December and the first week of January.

### Dose–response relationships for some epidemiologic indicators

**Figure 5:**
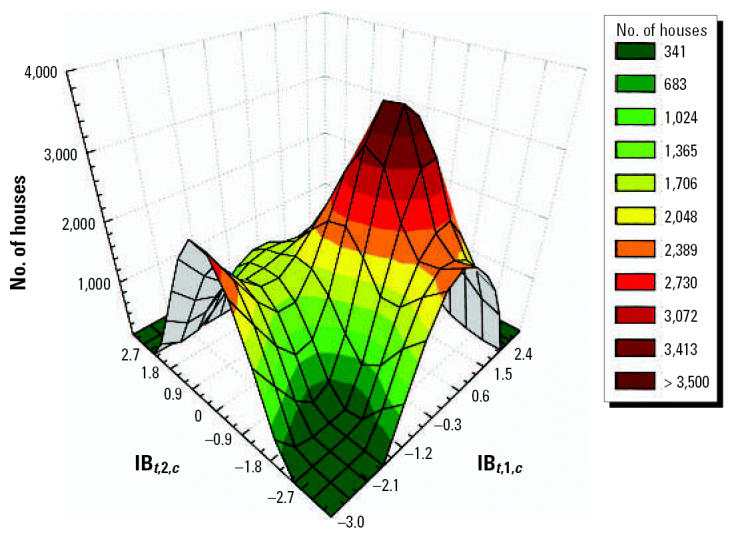
Association between CV and the number of houses with *A. aegypti* mosquitoes using the IB*_t,_*_1_*_,c_* and IB*_t,_*_2_*_,c_* indices.

**Figure 6:**
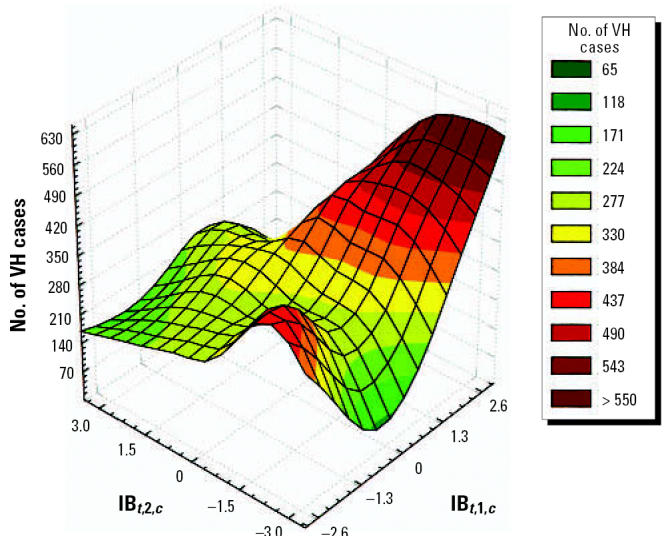
Association between CV and VH using the IB*_t,_*_1_*_,c_* and IB*_t,_*_2_*_,c_* indices.

Our results suggest that the incidences of VH and ADDs are associated with high levels of climatic anomalies. Table 1 presents stratified dose–response functions that can be used to estimate disease incidence for all geographic levels. The precision of the estimates depends on the disease, climate index, and coefficients for each geographic region or local area. Figure 5 shows the association between CV, based on the indices IB*_t_*_,1,_*_c_* and IB*_t_*_,2,_*_c_*, and the number of houses positive for *A. aegypti*. Figure 6 shows the association between CV and VH.

### Scenarios of changing CV

To create scenarios of CV, we divided Cuba into geographic regions: western region (including the provinces Pinar del Río, La Habana, Matanzas, and Juventud Island), central region (including the provinces Cienfuegos, Villa Clara, Sancti Spíritus, Ciego de Avila, and Camaguey), eastern region (including the provinces Las Tunas, Granma, Santiago de Cuba, and Guantánamo), 18 subregions, and eight zones according to the structure of the relief and the characteristics described by IB*_t_*_,_*_r_*_,_*_p_*.

We analyzed baseline data for 1961–1990 by decade and compared the data with conditions from 1991 to 2000. This allowed identification of CV in the different regions of the country. We found that major variability was related to the anticyclone. CV was found in the mean latitudes during the winter and to a lesser degree in mountainous regions and Juventud Island. We found less variability inland, in the eastern region, and along the southern coast (Ortíz Bultó and Rivero 2003b; Ortíz Bultó et al. 2004).

**Figure 7:**
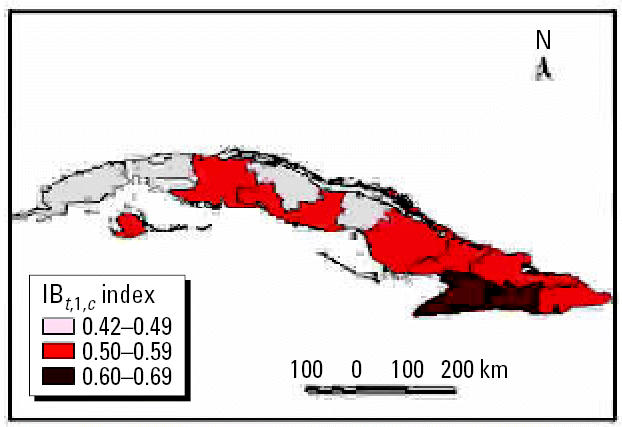
Projected anomalies in CV < 0.70 per decade using the IB*_t,_*_1_*_,c_* index.

**Figure 8:**
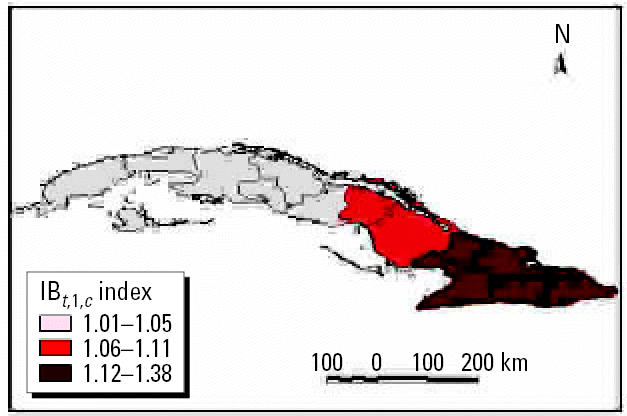
Projected anomalies in CV > 0.70 per decade using the IB*_t,_*_1_*_,c_* index.

**Figure 9:**
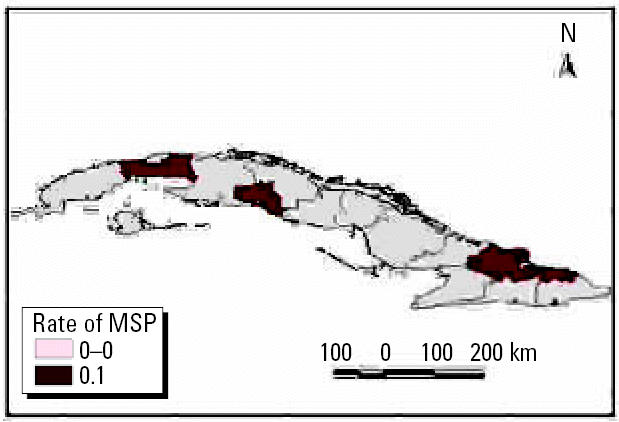
Projected incidence per 100,000 inhabitants of *S. pneumoniae* meningitis (MSP).

We performed analyses from the rainier period to the less rainy using all possible combinations. The results were used to describe and quantify the magnitude of CV in space–time using complex climatic indices. CV was stratified based on historic information on the effects of QBO and NAO, certain phases of which increase hot and dry weather during the warm season (Cárdenas 1998; Enfield 1998). ENSO results in more warm and rainy conditions during the cold season (Ortíz Bultó and Rivero 2004). The different combinations of CV resulted in the following scenarios: *a*) positive values of NAO with MEI in the warm phase and west–east QBO and *b*) negative values of NAO with the other parameters constant, for example, dry season described by IB*_t_*_,_*_i_*_,_*_c_* (Figures 7 and 8 show two extreme scenarios of variability with different levels of anomalies). Figure 9 shows the projected incidence rate for pneumococcal meningitis using the low-CV scenarios.

Using this type of analysis offers one tool for the development of surveillance systems to identify control and/or adaptation activities to reduce projected health impacts (Table 2).

### Climate change scenarios

**Figure 10:**
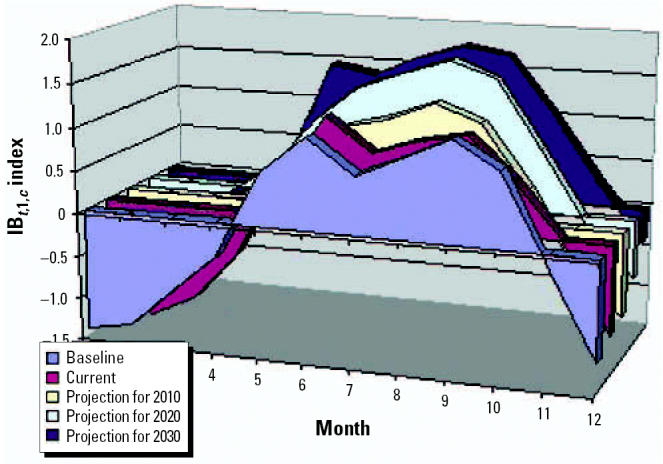
Climate change scenarios using the IB*_t_*_,1,_*_c_* index.

Climate scenarios were based on the HadCM2 (Hadley Center model) general circulation model using preindustrial carbon dioxide concentrations. The outputs were used to obtain CV rates (Mitchell et al. 1995) that were used as input to the Bultó indices (Ortíz Bultó et al. 1998; Ortíz Bultó and Rivero 2004). Climate change scenarios in 2010, 2020, and 2030 are shown in Figure 10.

**Figure 11:**
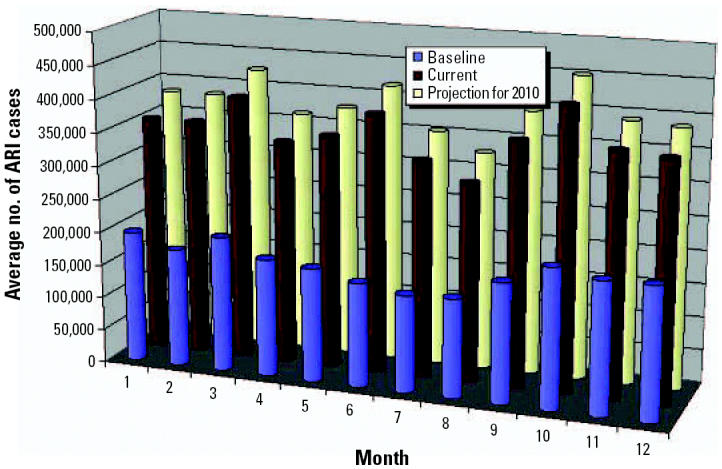
Projected impacts of climate change on ARIs.

**Figure 12:**
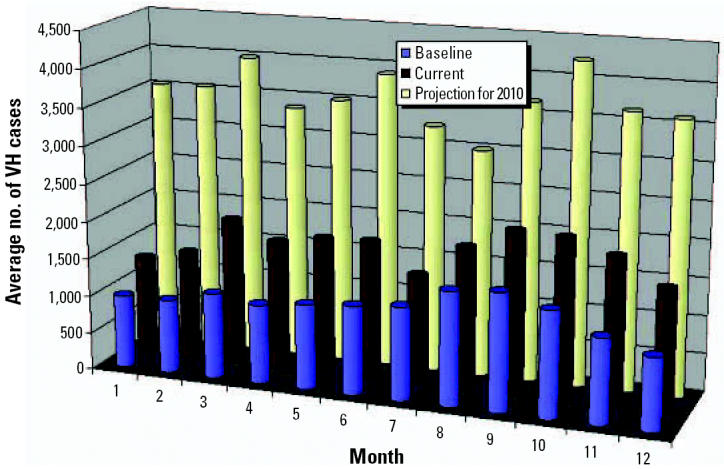
Projected impacts of climate change on VH.

Under both scenarios, the projected climate conditions are associated with an increase of ARIs and VH by oral/food transmission in 2010 (Figures 11 and 12). For ARIs, a new outbreak is projected for June. For VH, an increase in incidence is projected in the first months of the year, reaching a maximum in March. Climate conditions in winter seasons are projected to be warmer and rainier, and the rainy season is projected to be drier and hotter, which may then influence the incidence of VH.

It is important to note that some Bultó indices suggest more impact than others based on the epidemiologic characteristics of the disease. Therefore, each health outcome is likely to respond differently to CV and climate change. It is important to understand that many factors can influence the rate and intensity of these diseases, such as the effectiveness of community response.

### Adaptation measures in Cuba

Whether the projected health impacts of CV and climate change are actually experienced will depend on the measures used to attenuate or prevent these impacts. Adaptation includes the strategies, policies, and measures designed and implemented to reduce potential adverse health effects. Increasing the adaptive capacity of a population shares similar goals with sustainable development, increasing the ability of individuals and communities to cope with changes and challenges (Burton and van Aalst 1999).

Experience in Cuba has shown that primary health care is a key level for the implementation of preventive measures to reduce population vulnerability, particularly when considering the multiple factors that are related to climate-sensitive diseases. In addition to strengthening these programs, it is important to strengthen the linkage of the health sector with other sectors.

Generally, the vulnerability of a population to climate-related health risks depends on important aspects of the local environment. The level of material resources, the effectiveness of the government and civil institutions, the quality of the public health infrastructure, and access to relevant local information on extreme weather threats (Haines and Patz 2004; Woodward et al. 1998) are essential for the development of effective adaptation responses to reduce current and future vulnerability in the community. It is necessary to identify and prioritize strategies, policies, and measures to address CV and climate change and variability, as shown in Table 3.

### Capacity and response

Cuba’s Civil Defense Organization is in charge of anticipatory (proactive adaptation) and control measures for disaster and other emergency situations; this organization collaborates with the Ministry of Public Health, the Ministry of Science Technology and Environment, and others. In addition, a scientific working group has been created that includes experts in different disciplines and sectors and that develops studies on climate and human health. This group is able to asssess the risk of seven communicable diseases. This information is published in the monthly epidemiologic bulletin of El Instituto de Medicina Tropical “Pedro Kourí” (IPK 2006), available on the IPK website, and provides all decision makers with appropriate information for health planning and the design of appropriate measures.

### Using climate forecasts to predict outbreaks of climate-sensitive diseases

**Figure 13:**
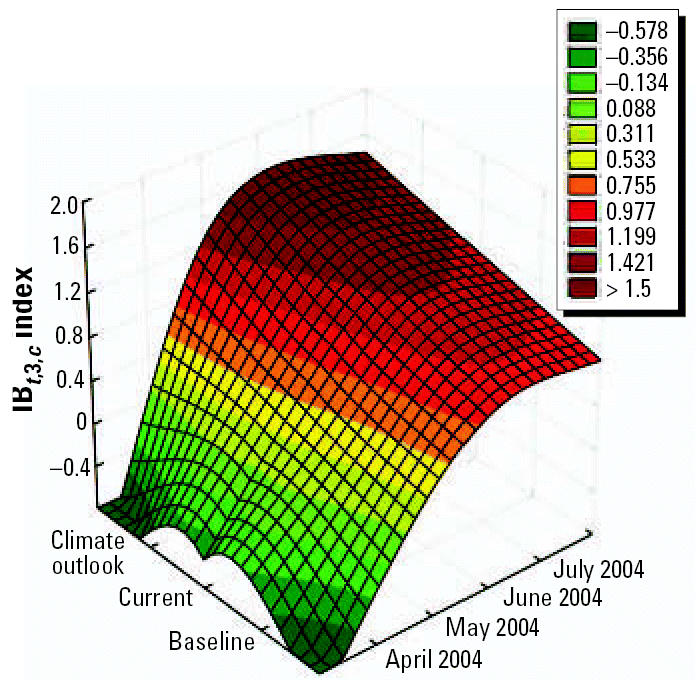
Baseline (1961–1990), current conditions (1991–2003), and climate outlook April–July 2004 using the IB*_t_*_,3,_*_c_* index.

**Figure 14:**
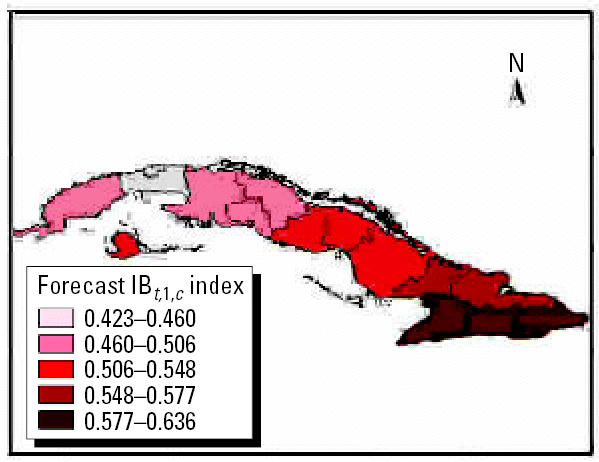
Climate outlook by region using the IB*_t_*_,1,_*_c_* index.

**Figure 15:**
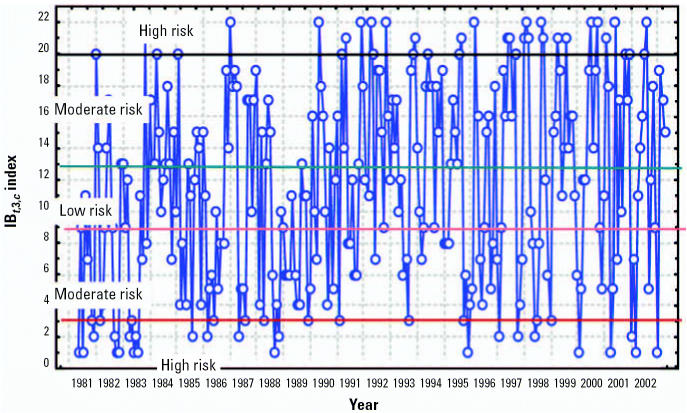
Risk levels using the IB*_t_*_,3,_*_c_* index.

**Figure 16:**
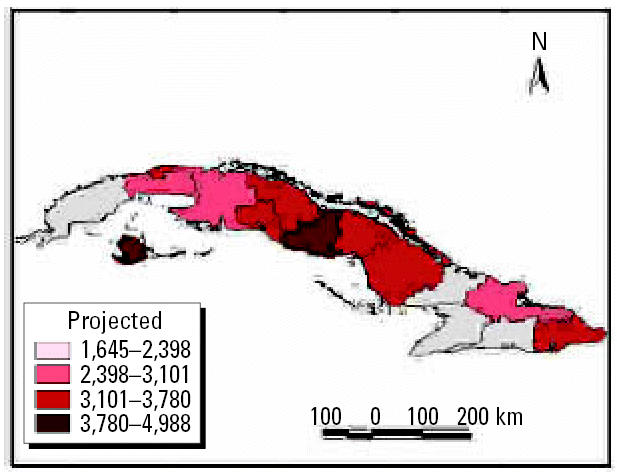
May 2004 projection for medical care for ARI (per 100,000 inhabitants).

**Figure 17:**
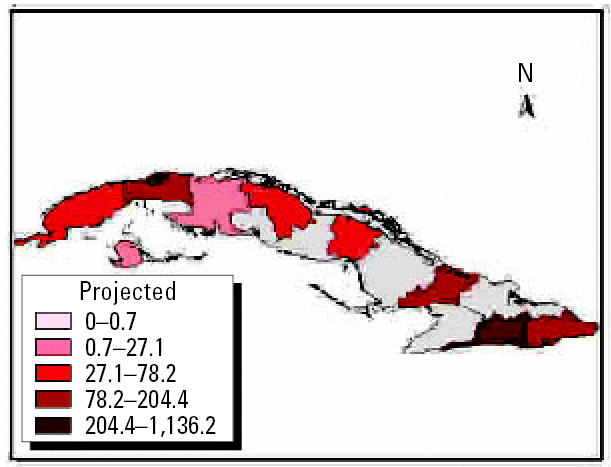
Projected cases per 100,000 inhabitants for ARI and dengue fever, May 2004.

Projections of disease outbreaks afford decision makers the opportunity to proactively initiate activities to reduce the impacts of outbreaks. Recent advances in seasonal forecasting are generating new opportunities to minimize the impact of CV on health [World Health Organization (WHO) 2004]. For this reason, using climatic indices along with forecasting models can alert authorities of possible changes in the risk level, either immediately or in the near future (Ortíz Bultó and Rivero 2003a). Further, this approach can be used to project how changing weather patterns might alter the range and intensity of climate-sensitive diseases. Figures 13–15 show the projections from the climate indexes as well as the risk level according to the IB*_t_*_,3,_*_c_*. The temporal risks for each region of the country can be projected by linking disease incidence with demographic data and the climate indices (Figures 13 and 14). Decision makers can use these results to plan anticipatory adaptation (proactive adaptation) measures, such as early warning systems (McMichael et al. 2003; WHO 2004). For example, under some climatic conditions, an increase in ADDs, ARIs, and the number of *A. aegypti* mosquitoes would be expected; the latter could result in a high risk for dengue transmission in the May to July period (Figures 16 and 17).

### Economic impacts of CV and climate change

Analyzing the economic impact of the effects of CV and climate change on human health is a complex and difficult undertaking. We used statistical data on the costs of hospitalization, treatments, and urgent care services to assess the economic impacts (McMichael et al. 2003). To estimate the costs of morbidity attributable to CV, we first needed to determine how many cases are attributable to CV. For each disease selected for analysis, we determined any changes in disease trends due to CV. We then determined levels of disease risk, including projected increased numbers of cases, using the dose–response functions, stratified on climate indices (Figure 15). Finally, the costs associated with excess cases over baseline were estimated (Tables 4 and 5).

## Conclusion

These results demonstrate that studies of climate and health are necessary to increase our knowledge of the effects of climate on human health; such information is important for decision makers and for reducing the economic–social impacts of CV and climate change. This study is innovative in the development of complex climate indices to reflect climate anomalies at different scales and to explain the mechanisms and relationships between climatic conditions and diseases. Our results suggest that some diseases not previously thought to be climate sensitive (VH, chicken pox, bacterial and viral meningitis, and others) vary with the identified climatic indices. The disease risks vary by geographic region, as described by the indices. Therefore, climate projections can be used to inform the design and development of prevention activities to reduce the burden of climate-sensitive diseases, thus increasing adaptive capacity to CV. Anticipatory prevention is better than reacting once a disease outbreak has occurred.

## Figures and Tables

**Table 1:** Impacts of CV on some diseases: dose–response functions.

Disease	Impact	Coefficient estimate (change in IB*_t_*_,3,_*_c_*)^a^
ADDs	High	1,109
	Medium	458.9
	Low	311.8
VH	High	31.42
	Medium	27.18
	Low	18.77

a This is multiplied by the susceptible population in the study region.

**Table 2:** Potential health impacts of high CV using the IB*_t_*_,1,_*_c_* index.

Disease	Trend	Effect
Airborne diseases		
BA	–	Decrease in the number of winter cases
ARIs	++	New epidemic peak in the warm season
Meningococcal disease	+	Increase in incidence in the winter season
Chicken pox	++	Advance of the epidemic peak
Water- and foodborne diseases		
VH	++	Increase in incidence in the winter season
ADD	++	Earlier increase in incidence in winter months
Vectorborne disease		
Dengue fever	++	More frequent epidemic outbreaks and a change in the season and spatial pattern

Symbols: –, projected decrease; +, projected small increase; ++, projected large increase.

**Table 3:** Some examples of adaptation measures to CV and climate change.

Adaptation options	Current activities	Future activities
Strengthen primary health care and the public health system	Specific health promotion and preventive programs designed to reduce population vulnerability Educational programs of environmental risks, including CV and climate change and their effects on human health	Continue developing health promotion and preventive programs, increasing community participation on health issues Increase the participation of local governments and other sectors in developing the best conditions of life
Measures to improve health surveillance systems	Provide forecasts of the main climate-sensitive diseases to all levels of the National Public Health System Increase number of early warning systems to predict epidemics	Continue research to improve forecast models using the necessary indices Incorporate new diseases and risk factors in the forecast models Decrease uncertainty through improved data and research on climate, epidemic, ecologic, and social variables
Immunization programs, particularly for high-risk groups	Maintain the current vaccination program and prioritize new programs	Enhance vaccination programs for ARIs and *Haemophilus influenzae* to achieve their successful control; maintain antimeningococcal immunization program; develop a prevention program for chicken pox
Improve sanitary conditions	Develop responses to increased sanitary demands in all fields (communal, drinking water, garbage, sewage, food, and others) Maintain contingency plans	Develop educational programs about environmental care with the participation of the community, government, and all sectors Increase environment care projects Improve contingency plans
Educational programs on radio and TV and in newspapers	Develop educational programs on the health risks associated with CV and change	Implement new programs on climate–health associations and communicate results to the population, governments, and others
Publish forecasts of communicable diseases through IPK Epidemiological Bulletins (IPK 2006)	Distribute the IPK Bulletin to all levels of the National Public Health System	Develop forecasts for each province and municipality
Exchange information with international researchers working on climate change and health issues	Participate in international meetings	Develop new projects with participation from other countries

**Table 4:** Estimated health care costs (US$) associated with CV, January 2001 through March 2002.

	Cost					
Disease	Health care visits	Hospitalization	Treatment	Urgent care	Work loss	Total
VH	8,874	8,657	5,505	1,237	91,750	116,023
ADD	373,074	175,068	76,065	36,463	547,059	1,207,729
Dengue fever	—	—	3,745,606	—	—	3,745,606
*Streptococcus pneumoniae* meningitis (hospitalized)	—	231,318	—	—	—	231,318
Total						5,300,675

**Table 5:** Projected economic costs (US$) of CV in 2010.

Disease	Projected no. of increased cases	Cost of increased cases	No. of hospitalizations	Hospitalization costs	Total cost
ADD	137,378	26,835,419	41,213	9,046,254	35,881,672
ARI	332,615	44,054,857	99,784	34,045,303	78,100,160
Dengue fever (hospitalized)	1,220	—	1,226,222	—	1,226,222
Meningococcal disease	3,001	—	3,001	2,400,800	2,400,800
*Streptococcus pneumoniae* meningitis (hospitalized)	100	—	100	814,500	814,500
Varicella	19,353	2,563,111	—	—	2,563,111
VH	11,027	1,433,510	3,308	1,966,838	3,400,348
Total					124,386,813
